# TECHNOLOGY TO SUPPORT AGING ADULTS WITH A COGNITIVE IMPAIRMENT

**DOI:** 10.1093/geroni/igad104.0316

**Published:** 2023-12-21

**Authors:** Sara Czaja, Lesley Ross

## Abstract

Technology applications can play a key role in enhancing independence and quality of life for aging adults with a cognitive impairment (CI) and offer potential benefits in terms of memory support, cognitive remediation, safety/security, and engagement. Drawing from research conducted by the Center for Research and Education on Aging and Technology Enhancement (CREATE) and the Center for Enhancing Neurocognitive Health, Abilities, Networks, & Community Engagement (ENHANCE) this symposium will highlight examples of existing and emerging technology applications that provide support for aging adults with a CI and discuss challenges and benefits with technology-based approaches. S. Czaja will present findings from a pilot trial that evaluated the benefits of a technology system designed to support information and resource access, and social and cognitive engagement (PRISM-CI) for aging adults with a CI. W. Boot will focus on the unique benefits of virtual reality (VR) for aging adults with and without a cognitive impairment and highlight gaps in knowledge regarding the use of VR to provide meaningful social and cognitive engagement. N. Charness will discuss a project (AUGMENT) that is focused on providing support for mobility for aging adults with a CI due to stroke, MCI, or a traumatic brain injury. W. Rogers will highlight how video technology can provide socially and cognitively engaging activities for aging adults with a CI and present a systematic process to help guide the development of a video intervention for this population. Lesley Ross will lead a discussion of the highlighted topics and areas for future research.

